# P-1210. Resurgence of Parechovirus-A3 Infection in Neonates and Young Infants after the Coronavirus Disease 2019 Pandemic in Japan

**DOI:** 10.1093/ofid/ofae631.1392

**Published:** 2025-01-29

**Authors:** Yuta Aizawa, Jun Tachikawa, Takayuki Yamanaka, Takayo Shoji, kenta Ito, Masashi Kasai, Hiroki Sakurai, Kisei Minami, Tadashi Hoshino, Meiwa Shibata, Kenji Kondo, Takanori Funaki, Yoshihiko Terauchi, Kenji Furuno, Yoshiaki Cho, Akihiko Saitoh

**Affiliations:** Niigata University Graduate School of Medical and Dental Sciences, Niigata, Niigata, Japan; Department of Pediatrics , Niigata University Graduate School of Medical and Dental Sciences, Niigata, Japan, Niigata, Niigata, Japan; Niigata City General Hospital, Niigata, Niigata, Japan; Shizuoka Children's Hospital, Shizuoka, Shizuoka, Japan; Aichi Children’s Health and Medical Center, Daifu, Aichi, Japan; Hyogo Prefectural Kobe Children’s Hospital, Kobe, Hyogo, Japan; Miyagi Children’s Hospital, Sendai, Miyagi, Japan; Nagano Children’s Hospital, Azumino, Nagano, Japan; Chiba Children’s Hospital, Chiba, Chiba, Japan; Division of Infectious Diseases, Department of Pediatrics, Tokyo Metropolitan Children’s Medical Center, Tokyo, Japan., Fuchu, Tokyo, Japan; NTT East Sapporo Hospital, Sapporo, Hokkaido, Japan; National Center for Child Health and Development, Tokyo, Tokyo, Japan; Kochi University, Kochi, Kochi, Japan; Fukuoka Children's Hospital, Fukuoka, Fukuoka, Japan; Okinawa Prefectural Nanbu Medical Center and Children’s Medical Center, Okinawa, Okinawa, Japan; Niigata University, Niigata, Niigata, Japan

## Abstract

**Background:**

Parechovirus-A (PeV-A), especially PeV-A3, is a common cause of viral sepsis and encephalitis in neonates and young infants (age < 4 months). The strict infection control measures and non-pharmaceutical interventions of the coronavirus disease 2019 (COVID-19) pandemic affected the epidemiological profiles of community-acquired diseases. However, the effects of the pandemic on the epidemiology of PeV-A infection in this age group are unknown.

**Methods:**

This prospective multicenter observational study was conducted between 2019 and 2023 in 12 Japanese prefectures. Neonates and young infants hospitalized for suspected sepsis and/or meningoencephalitis were included. Samples were sent to the laboratory of Niigata University, and PeV-A was detected by real-time PCR. The 7 hospitals introduced the BioFire Filmarray meningitis/encephalitis (FA/ME) panel in 2020. Genotype was determined by direct sequencing and BLAST analysis, and the phylogenetic tree of viral protein 1 region of PeV-A3 was constructed.

**Results:**

131 patients with PeV-A infection were identified (n = 36 in 2019, n = 7 in 2020, n = 1 in 2021, n = 27 in 2022, and n = 60 in 2023). Genotype was PeV-A3 in 124 cases and PeV-A4 in 2 cases in 2022 and unknown in 5 cases in 2019. Seasonal peaks and other characteristics were similar before and after the COVID-19 pandemic (i.e., in 2019 vs. 2023). Body temperature, fever duration, and the proportion of patients with rash declined; however, the proportion of those with abdominal distention increased. The FA/ME panel was more frequently used after the start of the pandemic (3.2% vs. 53.3%, *P* < 0.001). The proportion of those with sepsis declined from 83.9% to 63.3% (*P* = 0.04), but the proportions of those with encephalitis and those requiring ICU admission were similar. No patient had sequelae at discharge. PeV-A3 has evolved within a single clade with two branches.

**Conclusion:**

The number of PeV-A3 infections was higher after the start of the COVID-19 pandemic, possibly because low PeV-A3 activity early in the pandemic increased the number of susceptible pregnant women, thus leading to a decline in maternally derived antibodies. In addition, use of the FA/ME panel might help detect mild cases.

**Disclosures:**

**All Authors**: No reported disclosures

